# A Case of Synovitis Acne Pustulosis Hyperostosis Osteitis (SAPHO) Syndrome With Myeloperoxidase Anti-neutrophil Cytoplasmic Antibody: Exploring an Association or Coincidence

**DOI:** 10.7759/cureus.83866

**Published:** 2025-05-10

**Authors:** Koichi Kimura, Koji Hayashi, Asuka Suzuki, Mamiko Sato, Yuka Nakaya, Naoko Takaku, Toyoaki Miura, Yasutaka Kobayashi

**Affiliations:** 1 Department of Rehabilitation Medicine, Fukui General Hospital, Fukui, JPN; 2 Department of Neurology, University of Fukui, Fukui, JPN; 3 Graduate School of Health Science, Fukui Health Science University, Fukui, JPN

**Keywords:** antineutrophil cytoplasmic antibody (anca), dwibs, idiopathic interstitial pneumonia, myeloperoxidase-anti-neutrophil cytoplasmic antibodies (mpo-anca), sapho syndrome, vitiligo vulgaris

## Abstract

We report the first documented case of synovitis, acne, pustulosis, hyperostosis, and osteitis (SAPHO) syndrome in a Japanese male with positive serum myeloperoxidase antineutrophil cytoplasmic antibodies (MPO-ANCA). The patient, who initially presented with a history of hypertension and cerebral hemorrhage at age 73, was admitted for respiratory symptoms and gastrointestinal issues. Laboratory tests revealed elevated MPO-ANCA levels (34.7 IU/mL), leading to a diagnosis of interstitial pneumonia (IP) based on chest imaging. Conservative management was initiated, and oral prednisolone (PSL) therapy (initially 20 mg/day) was introduced at age 75 due to increased C-reactive protein (CRP) levels. However, the MPO-ANCA levels recorded two and five months after therapy were 52.8 and 71.8 IU/mL, respectively.

At age 76, he developed weakness in the right lower limb and gait disturbance following a stroke. Admission findings included elevated CRP levels (3.70 mg/dL) and a fresh infarction in the left corona radiata. Despite persistently elevated CRP, rehabilitation commenced. Follow-up imaging two months post-stroke showed new interstitial changes consistent with usual interstitial pneumonia (UIP) and sternoclavicular joint abnormalities suggestive of arthritis. The patient later developed a fever and a markedly high CRP level (19.91 mg/dL), prompting a resumption of PSL therapy (initially 60 mg/day). Post-treatment, interstitial pneumonia activity was controlled, and MPO-ANCA levels decreased to 3.2 IU/mL. The final diagnosis of SAPHO syndrome was established based on sternoclavicular arthritis and inflammatory changes.

While MPO-ANCA is primarily linked to autoimmune vasculitis, which can occasionally be accompanied by IP, the presence of MPO-ANCA in this case of SAPHO syndrome raises questions about its chance occurrence or potential association. This case highlights the first reported occurrence of SAPHO syndrome associated with MPO-ANCA positivity and underscores the need for further research to explore the relationship between autoimmune markers like MPO-ANCA and SAPHO syndrome.

## Introduction

Synovitis, acne, pustulosis, hyperostosis, and osteitis (SAPHO) syndrome, a condition combining musculoskeletal and dermatological symptoms, is rare and not fully understood. Its hallmark features include synovitis, acne, pustulosis, hyperostosis, and osteitis, typically present in cycles of flare-ups and remission [1-4]. The underlying causes remain unidentified, and the lack of standardized treatment options is a notable challenge [1,5]. Management often involves personalized approaches utilizing non-steroidal anti-inflammatory drugs (NSAIDs), bisphosphonates, and disease-modifying antirheumatic drugs (DMARDs) [6]. Nonetheless, symptom relief varies considerably among individuals, frequently necessitating a combination of treatments [3,7-8]. Diagnosis remains complicated, as universally accepted criteria are unavailable, making it difficult to distinguish SAPHO from conditions like chronic non-bacterial osteomyelitis [9].

Myeloperoxidase (MPO) antineutrophil cytoplasmic antibodies (MPO-ANCA), also known as perinuclear anti-neutrophil cytoplasmic antibodies (p-ANCA), are commonly observed in various autoimmune and inflammatory diseases, most notably in ANCA-associated vasculitis (AAV). Among these, microscopic polyangiitis (MPA) and eosinophilic granulomatosis with polyangiitis (EGPA) are frequently associated with MPO-ANCA, which typically targets MPO as its autoantigen [10,11]. MPO-ANCA positivity is also reported in systemic autoimmune diseases such as systemic lupus erythematosus (SLE) [12] and in rheumatoid arthritis-associated interstitial lung disease (RA-ILD), where it correlates with more severe respiratory symptoms and poorer lung function [13]. Furthermore, patients with idiopathic interstitial pneumonia (IIP) or interstitial pneumonia (IP) with autoimmune features (IPAF) can also present with MPO-ANCA positivity, and such individuals have a higher risk of evolving into systemic vasculitis over time [14].

This report describes a unique case of SAPHO syndrome accompanied by MPO-ANCA, a previously undocumented association that expands our understanding of this complex disorder.

## Case presentation

**Figure 1:**
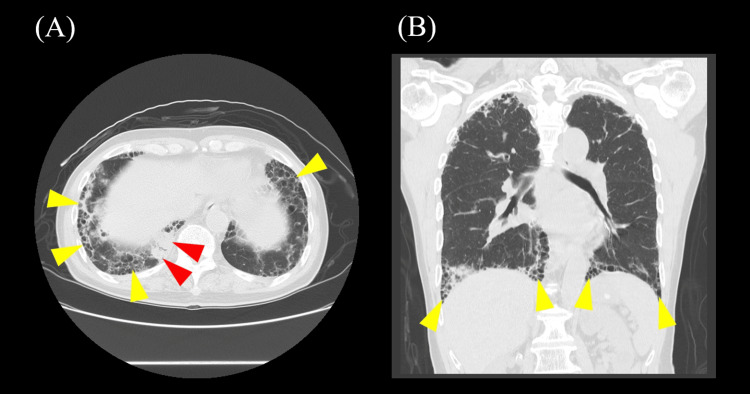
Results of the chest-abdominal computed tomography (CT) at age 73. (A, B) Chest-abdominal CT showing subpleural interstitial opacities and honeycombing in both lungs (yellow arrowhead), infiltrates in the right lung base (red arrowhead). (A) Axial section; (B) coronal section.

A 73-year-old Japanese male with a medical history of hypertension, hyperlipidemia, appendicitis in his 40s, cerebral hemorrhage at age 54, and cerebellar infarction at age 64 presented to our hospital with cough, fever, vomiting, and diarrhea. The COVID-19 antigen test was negative. Blood tests revealed elevated levels of white blood cells, neutrophils, lactate dehydrogenase (LDH), C-reactive protein (CRP), aspartate aminotransferase (AST), KL-6, and surfactant protein D (SP-D), along with decreased levels of red blood cells, hemoglobin, hematocrit, lymphocytes, and eosinophils (Table 1). Serum autoantibody markers, including antinuclear antibody, anti-SS-A antibody, and anti-SS-B antibody, were negative, but elevated MPO-ANCA of 34.7 IU/mL (reference range: <3.4 IU/mL) was observed (Table 2). Chest-abdominal computed tomography (CT) revealed subpleural interstitial opacities and honeycombing in both lungs, infiltrates in the right lung base (Figure 1). Based on these findings, he was diagnosed with interstitial pneumonia (IP). As his clinical condition remained stable, he was managed conservatively and followed as an outpatient without initiating specific treatment. He underwent routine follow-up with chest CT scans every six months, which showed no significant progression of IP.

**Table 1:** Blood test results at age 73. eGFR, estimated glomerular filtration rate

Inspection items	Result	Reference
Red blood cell (RBC)	429 × 10⁴/μL	435-555 × 10⁴
White blood cell (WBC)	15,800/μL	3,300-8,600
WBC differential %		
Neutrophils	90.10%	40.0-70.0
Lymphocytes	6.30%	25.0-45.0
Monocytes	3.20%	2.0-7.0
Eosinophils	0.30%	1.0-6.0
Basophils	0.10%	0.0-1.0
Hemoglobin	13.3 g/dL	13.7-16.8
Platelet (PLT)	16.6 × 10⁴/μL	15.8-34.8 × 10⁴
Total protein (TP)	8.0 g/dL	6.6-8.1
Albumin (Alb)	4.1 g/dL	4.1-5.1
Blood urea nitrogen (BUN)	20.0 mg/dL	8.0-20.0
Creatinine (Cre)	0.91 mg/dL	0.65-1.07
Total bilirubin (T-Bil)	0.6 mg/dL	0.4-1.2
Aspartate aminotransferase (AST)	32 U/L	13.0-30.0
Alanine aminotransferase (ALT)	18 U/L	10.0-30.0
Alkaline phosphatase (ALP) (IFCC)	109 U/L	38.0-113.0
Alkaline phosphatase (ALP) (JSCC)	310 U/L	106.0-322.0
Lactate dehydrogenase (LDH)	321 U/L	124.0-222.0
Amylase	109 U/L	44.0-132.0
Sodium (Na)	140 mmol/L	138.0-145.0
Potassium (K)	3.9 mmol/L	3.6-4.8
Chloride (Cl)	104 mmol/L	101.0-108.0
Calcium (Ca)	9.8 mg/dL	8.8-10.1
eGFR	62.8 mL/minute/1.73 m²	>60
C-reactive protein (CRP)	2.85 mg/dL	0.00-0.14
KL-6	503 U/mL	0-499
Surfactant protein D (SP-D)	171.3 ng/mL	0-109.9

**Table 2:** Serum autoantibody marker results at age 73. RIA, radioimmunoassay

Inspection items	Results	Reference range
Rheumatoid factor (RF)	96.7 IU/mL	0-15
Antinuclear antibody (ANA)	<1:40
Anti-DNA antibody (RIA method)	3.7 IU/ｍL	0.0-6.0
Anti-RNP antibody	1.6 U/mL	<3.4
Anti-Sm antibody	1.2 U/mL	<6.9
Anti-Sjögren’s syndrome-related antigen A antibody (Anti-SSA antibody)	0.5 U/mL	<6.9
Anti-Sjögren’s syndrome-related antigen B antibody (Anti-SSB antibody)	0.4 U/mL	<6.9
Anti-Topoisomerase I antibody (Anti-Scl-70 antibody)	<0.6 U/mL	<6.9
Anti-histidyl-tRNA synthetase antibody (Anti-Jo-1 antibody)	<0.3 U/mL	<6.9
Anti-centromere antibody	0.4 U/mL	<6.9
Proteinase-3-antineutrophil cytoplasmic antibody (PR3-ANCA)	<0.6 IU/mL	<1.9
Myeloperoxidase anti-neutrophil cytoplasmic antibody (MPO-ANCA)	34.7 IU/mL	<3.4
Anti-aminoacyl-tRNA synthetase antibody (Anti-ARS antibody)	<5.0 IU/mL	<24.9

Since the age of 74, he had been suffering from chronic eczema on his limbs and trunk, requiring topical corticosteroid treatment. The skin lesions were itchy, with marked lichenification and excoriation. He also complained of occasional coughing, which was chronically treated with antitussives.

At age 75, oral prednisolone (PSL) therapy was initiated at an initial dose of 20 mg/day due to exacerbations of coughing and elevated CRP levels, suspecting an exacerbation of IP. Steroid treatment significantly reduced CRP levels. PSL was tapered and then discontinued over two months. Unfortunately, although we did not measure MPO-ANCA levels immediately before or after steroid treatment, the MPO-ANCA levels recorded two and five months after therapy were 52.8 and 71.8 IU/mL, respectively.

**Figure 2:**
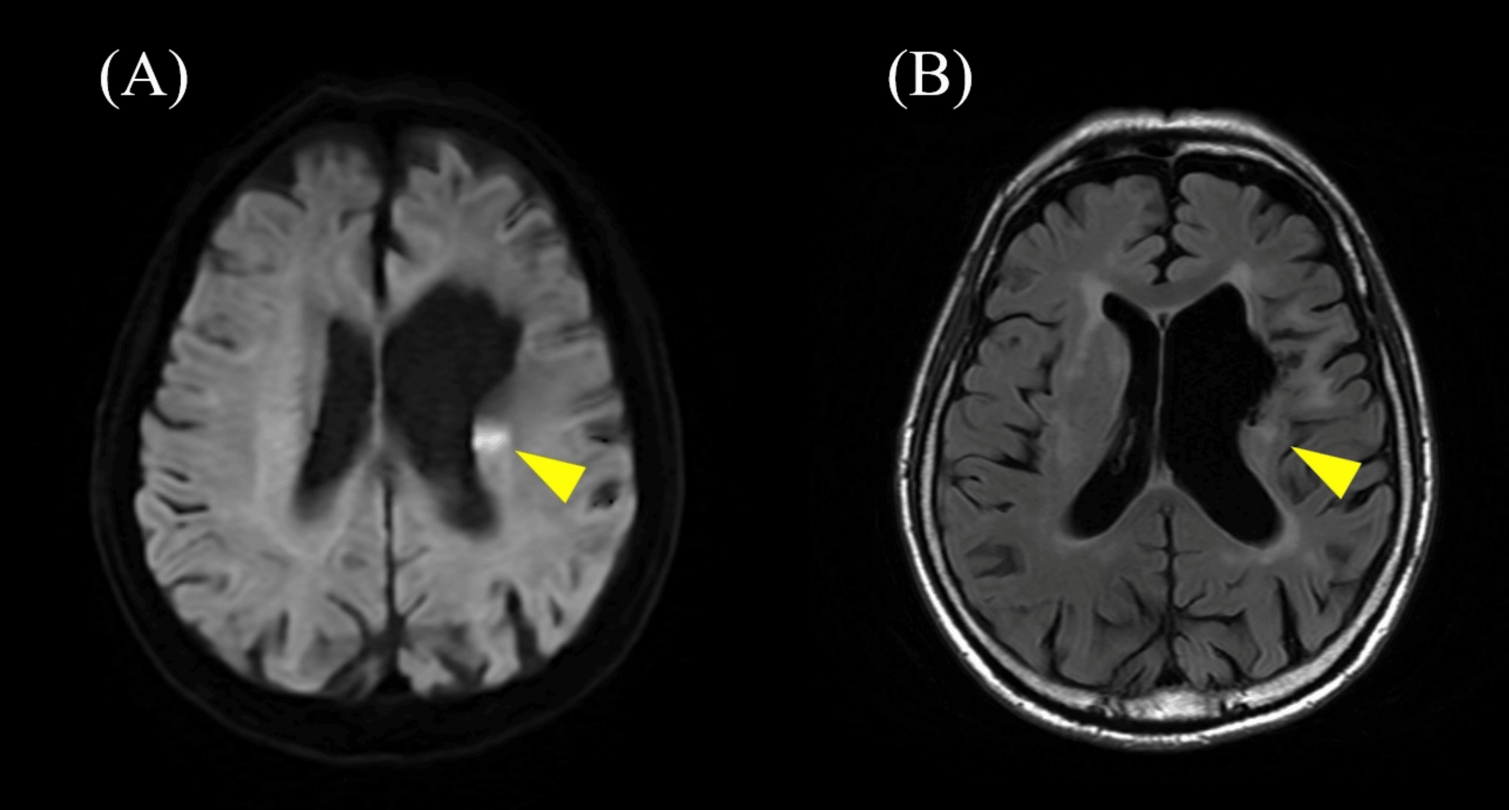
Brain magnetic resonance imaging (MRI) at stroke onset (obtained at a previous hospital) at age 76. Brain MRI showing a hyperintensity area in the left corona radiata (arrowhead): (A) diffusion-weighted imaging (DWI); (B) T2-fluid attenuated inversion recovery imaging (T2-FLAIR).

At the age of 76, during a routine checkup just before hospitalization, MPO-ANCA was found to be elevated at 99.0 IU/mL. Half a month later, he developed weakness in the right lower limb and gait disturbance, prompting a visit to a previous hospital. Brain magnetic resonance imaging (MRI) revealed a fresh infarction in the left corona radiata (Figure 2), and he was treated with antiplatelet and anticoagulant therapy as an acute-phase treatment. One month after stroke onset, he was transferred to our hospital for rehabilitation. On admission, a dry cough was noted. Vital signs showed body temperature of 36.5 °C, blood pressure of 136/84 mmHg, and heart rate of 87 beats per minute. General physical examinations revealed fine crackles in both lungs and vitiligo on the right side of the abdomen. Neurological examinations revealed mild weakness in the right leg and a dragging gait. Cognitive assessments indicated acalculia, impaired memory, and reduced attention span. Blood tests revealed a significantly elevated white blood cell count of 108 × 10²/µL, D-dimer level of 2.1 μg/mL, alkaline phosphatase (ALP) level of 164 U/L, and C-reactive protein (CRP) level of 3.70 mg/dL, along with decreased levels of hemoglobin at 12.1 g/dL, hematocrit at 37.10%, albumin at 2.5 g/dL, and high-density lipoprotein (HDL) cholesterol at 37 mg/dL (Table 3). Although inflammatory markers, including CRP, were elevated, the patient had no clinical symptoms such as fever, allowing rehabilitation treatment to commence without any issues, and his activities of daily living (ADL) gradually improved.

**Table 3:** Blood test results at the time of transfer to our hospital at age 76.

Inspection items	Result	Reference range
Red blood cells (RBC)	402×10⁴/μL	435-555
White blood cells (WBC)	108×10^2^/μL	33-86
Hemoglobin	12.1 g/dL	13-16
Hematocrit	37.10%	40-50
Mean corpuscular volume (MCV)	92.3 fL	83-98
Mean corpuscular hemoglobin (MCH)	30.1 pg	27-33
Mean corpuscular hemoglobin concentration (MCHC)	32.6 g/dL	31-35
Platelets	20.4×10⁴/μL	15-34
Prothrombin time (PT)	9.8 sec	9-13
Prothrombin activity	119.20%	70-130
Prothrombin time-international normalized ratio (PT-INR)	0.91	<1.0
Activated partial thromboplastin time (APTT)	26.1 sec	24-34
D-Dimer	2.1 μg/mL	<1.0
Blood glucose	82 mg/dL	73-109
HbA1c (NGSP)	5.50%	4.0-6.0
Total protein	7.3 g/dL	6.6-8.1
Albumin	2.5 g/dL	4.1-5.1
Blood urea nitrogen (BUN)	15.6 mg/dL	8-20
Creatinine	0.91 mg/dL	<1.0
Uric acid	4.4 mg/dL	3.7-7.0
Total bilirubin	0.2 mg/dL	0.4-1.2
Direct bilirubin	0.1 mg/dL	0.0-0.3
Indirect bilirubin	0.1 mg/dL	0.2-0.8
Aspartate aminotransferase (AST)	21 U/L	13-30
Alanine aminotransferase (ALT)	14 U/L	10-30
Alkaline phosphatase (ALP)	164 U/L	38-113
Lactate dehydrogenase (LDH)	139 U/L	124-222
Leucine aminopeptidase (LAP)	62 U/L	30-80
Gamma-glutamyltransferase (γ-GTP)	72 U/L	13-64
Creatine phosphokinase (CPK)	10 U/L	59-248
Cholinesterase	149 U/L	240-486
Amylase	103 U/L	44-132
Sodium (Na)	138 mmol/L	138-145
Potassium (K)	4.3 mmol/L	3.6-4.8
Chloride (Cl)	103 mmol/L	101-108
Calcium (Ca)	8.4 mg/dL	8.8-10.1
Triglycerides	89 mg/dL	40-149
High-density lipoprotein cholesterol (HDL cholesterol)	37 mg/dL	40-90
Low-density lipoprotein (LDL) cholesterol	65 mg/dL	65-139
Total cholesterol	120 mg/dL	142-219
eGFR	62.1 mL/min/1.73m²	60.0
C-reactive protein	3.7 mg/dL	0.00-0.14

**Figure 3:**
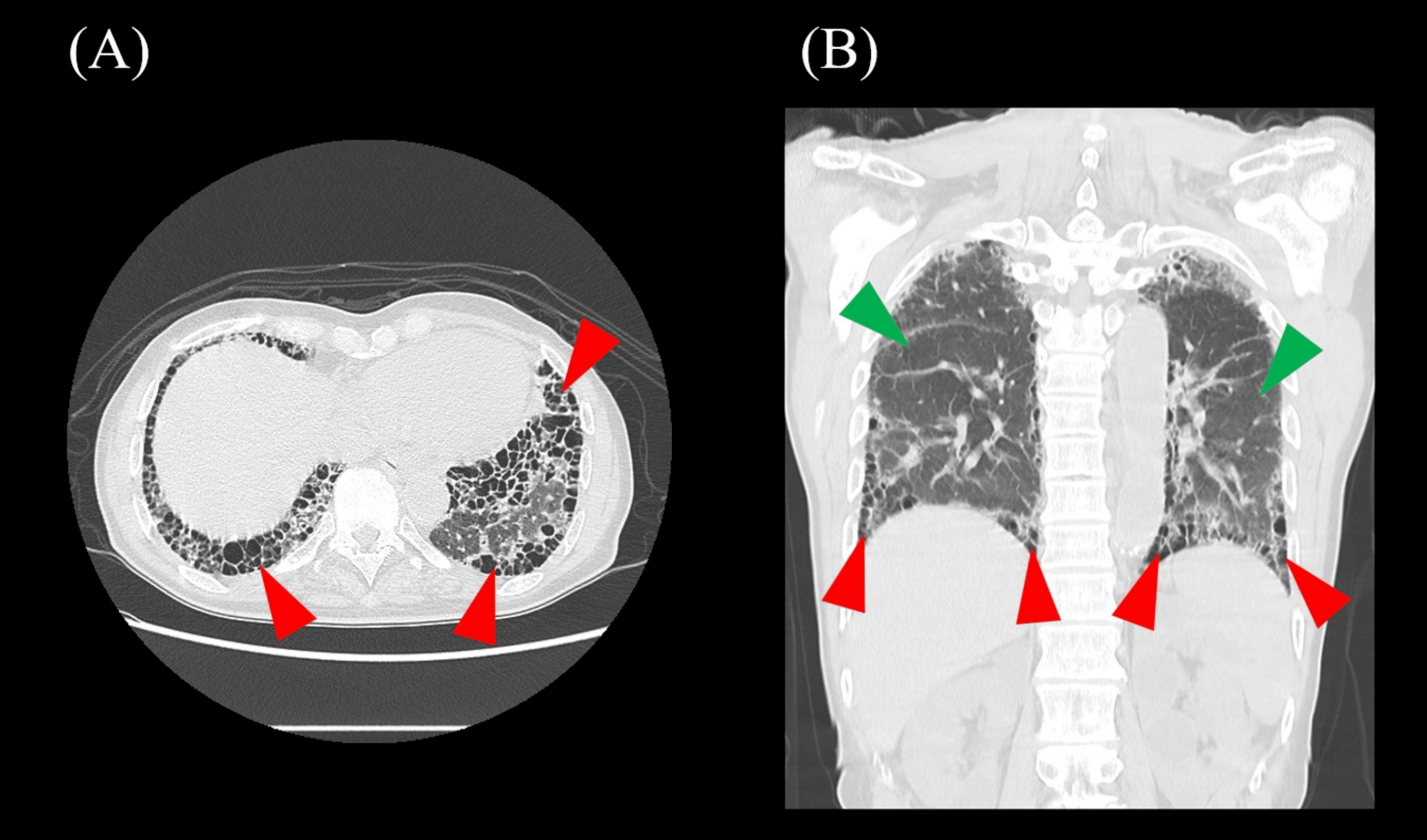
Results of the chest computed tomography (CT) two months after stroke. (A, B) Chest CT showing interstitial opacities predominantly distributed in the peripheral regions of both lungs, accompanied by honeycombing (red arrowheads), suggesting interstitial pneumonia with a usual interstitial pneumonia (UIP) pattern. Overall, decreased aeration and increased lung density are observed (green arrowheads), raising the possibility of acute exacerbation. (A) Axial section; (B) coronal section.

**Figure 4:**
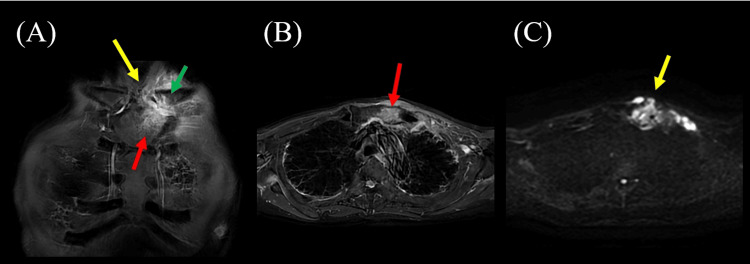
Results of the chest magnetic resonance imaging (MRI) two months after stroke. (A, B, C) Chest MRI showing soft tissue signal around and within the left sternoclavicular joint (yellow arrows). High signal intensity is also observed in the sternum (red arrow) and clavicle (green arrow). (A) Coronal section (short τ inversion recovery [STIR]); (B) axial section (short τ inversion recovery [STIR]); (C) axial section (diffusion-weighted imaging [DWI]).

Two months after the onset of the stroke, he became increasingly short of breath during rehabilitation exercises. Suspecting worsening IP, blood was taken, and the white blood cell count was found to be 99×10^2^/µL and CRP 3.8 mg/dL. Chest CT was performed, revealing new bilateral subpleural interstitial changes with honeycombing (Figure 3), consistent with usual interstitial pneumonia (UIP), and reduced lung transparency suggesting acute exacerbation. A chest MRI was conducted, which showed soft tissue signals within and around the left sternoclavicular joint, as well as signal changes in the sternum and clavicle, indicative of sternoclavicular arthritis (Figure 4). PSL 60 mg/day was initiated to treat exacerbation of IP and arthritis, and was gradually tapered. After starting steroids, the patient's cough was alleviated, and he experienced improvement in shortness of breath. Blood tests four weeks after starting PSL showed that CRP had dropped to 0.26 mg/dL and MPO-ANCA became negative at 3.2 IU/mL.

**Figure 5:**
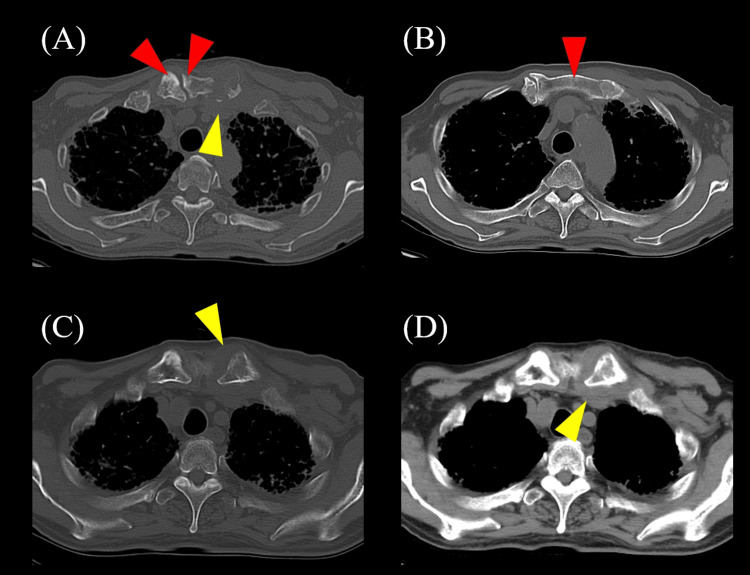
Results of the chest computed tomography (CT) five months after stroke onset. (A, B, C) Chest CT showing joint capsule thickening, joint effusion (yellow arrowheads), and signal changes in the sternoclavicular joint and surrounding bone: sternum and clavicle (red arrowheads).

**Figure 6:**
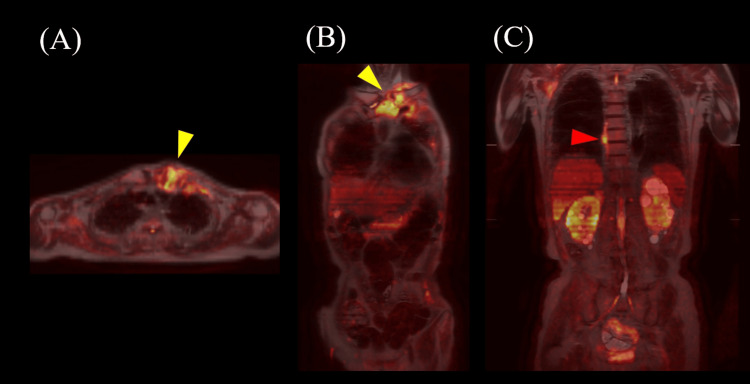
Results of magnetic resonance imaging (MRI) using diffusion-weighted whole-body imaging with background body signal suppression (DWIBS), obtained five months after stroke onset. (A, B, C) DWIBS showing hyperintensities in the left sternoclavicular joint (yellow arrowheads) and along the right edge of the T8 to T9 vertebral bodies (red arrowhead). (A) Axial section; (B, C) coronal sections.

Five months after the onset of the stroke, he developed anterior chest pain and a fever of 38.0 °C. Examination revealed swelling at the left sternoclavicular joint, and blood tests showed an elevated CRP level of 19.6 mg/dL. Chest CT revealed further findings of joint capsule thickening and joint effusion in the sternoclavicular joint, corroborating the diagnosis of sternoclavicular arthritis (Figure 5). Diffusion-weighted whole-body imaging with background body signal suppression (DWIBS) revealed hyperintensities in the left sternoclavicular joint and along the right edge of the T8 to T9 vertebral bodies (Figure 6). He was diagnosed with SAPHO syndrome and was transferred to his previous hospital for further management of the condition.

## Discussion

This report describes a case of SAPHO syndrome with serum MPO-ANCA positivity. He had persistently elevated inflammatory responses of unknown etiology, periosteitis, osteitis, osteosclerosis, chronic skin rash, as well as interstitial pneumonia with UIP pattern and vitiligo vulgaris. Unfortunately, there are no preserved photos of the skin lesions, and the description in the medical records regarding the skin lesions was insufficient; therefore, we were unable to make a diagnosis of acne or pustulosis. MPO-ANCA decreased markedly with steroid treatment. MRI on DWIBS revealed hyperintense lesions within the sternoclavicular joint, surrounding bones, overlying skin, and the right edge of vertebral bodies. While DWIBS is not yet widely adopted, it is a valuable MRI sequence that holds promise for the detection of inflammatory diseases [15,16].

The diagnosis of SAPHO syndrome is primarily clinical and radiological, as no specific diagnostic marker exists [17-21]. It should be suspected when inflammatory arthritis and/or osteitis, particularly involving the anterior chest wall, sacroiliac joints, or spine, is associated with a neutrophilic or acneiform dermatosis or psoriasis [17,18]. Several diagnostic criteria have been proposed, including those by Benhamou et al. and Kahn et al., and the presence of only one of the inclusion criteria is sufficient for making the diagnosis, but none are universally validated [9,20-22]. Benhamou et al.'s criteria include osteoarticular manifestations of severe acne, palmoplantar pustulosis (PPP), hyperostosis (of the anterior chest wall, limbs, or spine) with or without dermatosis, and chronic recurrent multifocal osteomyelitis (CRMO) with or without dermatosis [20,23,24]. Kahn et al. proposed their diagnostic criteria in 1994 [9] and modified them in 2003 [21]. Specifically, for diagnosis based on Kahn et al.’s criteria from 1994, at least one characteristic must be met among the following three features: (1) chronic recurrent multifocal sterile axial osteomyelitis, with or without dermatosis; (2) acute, subacute, or chronic arthritis associated with PPP, pustulous psoriasis, or severe acne; and (3) any sterile osteitis associated with PPP, pustulous psoriasis, or severe acne [9]. In 2003, they emphasized the association of bone/joint involvement with PPP and psoriasis, as well as severe acne, isolated sterile hyperostosis/osteitis in adults, CRMO in children, or bone/joint involvement with chronic bowel diseases [18,21,23].

Our case presented with symptoms including hyperostosis, chronic osteitis, sternoclavicular arthritis, and a history of chronic skin lesions on the limbs and trunk. In addition to the sternoclavicular joint and bone lesions, lesions across multiple vertebral bodies were detected by DWIBS. Although our patient had a history of skin lesions on the trunk and limbs that were treated with steroids, there were no characteristic PPP. Additionally, it was unclear from the medical records whether this rash was acne or pustular. Nonetheless, the patient exhibited hyperostosis and sterile multifocal osteomyelitis in the vertebrae and sternum, which met the diagnostic criteria set forth by Benhamou et al. and Kahn’s diagnostic criteria from both 1994 and 2003. Therefore, we made a definitive diagnosis of SAPHO syndrome.

Our case presented with SAPHO syndrome, accompanied by persistent MPO-ANCA positivity and interstitial pneumonia. While no diagnostic-specific markers exist for SAPHO syndrome, several markers associated with other autoimmune diseases have been reported to be positive in patients with SAPHO syndrome. One such marker is the anti-SSA and anti-SSB antibody associated with Sjögren's syndrome. According to past reports, the prevalence of Sjögren's syndrome in patients with SAPHO syndrome is 3.05%, with anti-SSA and anti-SSB antibodies being positive in 20% of diagnosed cases [25]. Anti-cyclic citrullinated peptide antibodies (anti-CCP antibodies), considered specific for rheumatoid arthritis (RA), were positive in 23.1% of patients with SAPHO syndrome [26]. Another study revealed that autoantibodies were found in 22.2% of patients with SAPHO syndrome; antinuclear antibodies (ANA) in 15.5% of patients; antithyroid antibodies, including anti-TPO and/or anti-Tg, in 3.3%; antigastric parietal cell antibodies in 3.3%; antismooth muscle antibodies in 4.4%; and anti-SSA antibodies in 2.3% [27].

To our knowledge, there are no reports in the English literature regarding ANCA positivity; however, we identified only one case report in the Japanese literature of SAPHO syndrome with PR3-ANCA positivity [28]. This patient presented with generalized pustular rash and sternoclavicular arthritis, consistent with an incomplete form of SAPHO syndrome. However, the patient also exhibited high fever, elevated inflammatory markers, conjunctivitis, episcleritis, retinal hemorrhage likely due to retinal vasculitis, and sensorineural hearing loss. Positive PR3-ANCA further supported a diagnosis of SAPHO syndrome with co-existing PR3-ANCA-associated vasculitis. Treatment with PSL 1 mg/kg resulted in rapid fever resolution, normalization of inflammatory markers, and improvement of symptoms.

Our case is positive for MPO-ANCA, which is the first report of its kind in association with SAPHO syndrome. The patient also exhibited chronic cough, cerebral infarctions, and an idiopathic UIP pattern. While we cannot entirely exclude the possibility of AAV or other ANCA-associated diseases, there was no definitive evidence to support this diagnosis. Furthermore, there are numerous reports of MPO-ANCA-positive IP associated with MPA and AAV [29], making it unclear whether the MPO-ANCA is a concurrent finding with SAPHO syndrome or an incidental occurrence.

As this represents the inaugural report on this topic, we cannot definitively determine whether the intriguing combination of SAPHO syndrome and MPO-ANCA is a true association or merely a coincidence. Nonetheless, this report promises to enhance our understanding of the clinical landscape of SAPHO syndrome and open new avenues for further research.

## Conclusions

This report presents the first documented case of SAPHO syndrome associated with serum MPO-ANCA positivity, highlighting a unique clinical profile characterized by persistent inflammatory responses, bone hyperplasia in the sternoclavicular joint, interstitial pneumonia with a UIP pattern, and vitiligo vulgaris. While past research has associated several autoantibodies with SAPHO syndrome, the presence of MPO-ANCA raises intriguing questions about its relevance in this context. Further investigation is needed to determine the relationship between SAPHO syndrome and MPO-ANCA. Ultimately, this report sets the stage for further exploration into the connections between autoimmune markers and SAPHO syndrome, paving the way for more comprehensive research in this area.
